# Risk and Protective Factors for Men’s Sexual Violence Against Women at Higher Education Institutions: A Systematic and Meta-Analytic Review of the Longitudinal Evidence

**DOI:** 10.1177/1524838020970900

**Published:** 2020-11-11

**Authors:** Bridget Steele, Mackenzie Martin, Alexa Yakubovich, David K. Humphreys, Elizabeth Nye

**Affiliations:** 1 Department of Social Policy and Intervention, University of Oxford, United Kingdom; 2 MAP Centre for Urban Health Solutions, Li Ka Shing Knowledge Institute, St. Michael’s Hospital, Unity Health Toronto, Ontario, Canada

**Keywords:** sexual assault, dating violence, domestic violence, offenders, sexual assault

## Abstract

Sexual violence among higher education institution (HEI) students is a growing public health concern. To date, there is little evidence on how to effectively prevent sexual violence among this demographic. This study is the first systematic review to meta-analyze all available evidence for risk and protective factors of sexual violence perpetrated by men at HEIs. We searched four electronic databases and multiple gray literature sources. We screened studies using prespecified selection criteria for the sample (HEI students who identify as men), outcome (sexual violence perpetration against peers), and study design (quantitative and longitudinal). Longitudinal studies provide the most rigorous available evidence on risk and protective factors. We identified 16 studies and meta-analyzed eight different risk factors: alcohol consumption, hostility toward women, delinquency, fraternity membership, history of sexual violence perpetration, rape myth acceptance, age at first sex, and peer approval of sexual violence. We deemed included studies to have a varied risk of bias and the overall quality of evidence to range from moderate to high. History of sexual violence perpetration (perpetration prior to entering an HEI) emerged as the strongest predictor of sexual violence perpetration at HEIs, complicating the notion that HEI environments themselves foster a culture of sexual violence. Peer support for sexual violence predicted perpetration while individual rape-supporting beliefs did not. Our findings suggest that interventions targeting peer norms (e.g., bystander interventions) and early sexual violence prevention and consent interventions for high school and elementary school students could be effective in reducing and preventing sexual violence at HEIs.

## Background

Peer-to-peer sexual violence at higher education institutions (HEIs)—organizations providing postsecondary or tertiary levels of education—is a serious public health concern (Mellins et al., 2017). Women attending HEIs face a higher risk of experiencing sexual violence when compared to men and when compared to women not attending HEIs (Bhochhibhoya et al., 2019; Degue et al., 2014; Gonzales et al., 2005; Zinzow & Thompson, 2015). A recent meta-analysis of research on sexual violence at HEIs in the United States estimates that over 20% of women have experienced unwanted sexual contact and that 0.5%–8.4% of women have experienced rape (Fedina et al., 2018). Many factors contribute to the challenge of establishing the prevalence of sexual violence in HEIs, most notably, the diversity of HEI settings (Duval et al., 2018), differing definitions and measurements of sexual violence (Anderson et al., 2019; Fedina et al., 2018; Mellins et al., 2017), and underreporting of sexual violence perpetration and victimization due to stigma, trauma, and a lack of understanding of what constitutes sexual violence (Mellins et al., 2017). Despite these issues with reporting, the literature consistently reveals that sexual violence at HEIs is a pervasive phenomenon (Bhochhibhoya et al., 2019).

### Sexual Violence and Dating Violence

It is worth examining sexual violence against women at HEIs (defined as any nonconsensual sexual experiences including rape, sexual assault, sexual aggression, and sexual harassment), independently from other forms of gender-based violence (World Health Organization [WHO], 2003). Dating violence or intimate partner violence are the terms most often used in literature discussing peer-to-peer sexual violence. The terms “dating” or “intimate partner” have limited relevance within an HEI context as they do not necessarily encompass the variety of behaviors and interactions occurring in this environment including “hookups” or casual relationships (Garcia et al., 2012).

In HEI settings, perpetrators can include classmates, friends, housemates, and acquaintances, as well as sexual partners and boyfriends (Flack et al., 2016). Existing research shows that the majority (60%) of young adults at HEIs in the United States have engaged in sexual acts with people they are not in a committed relationship with (Duval et al., 2018; Kuperberg & Padgett, 2016; Monto & Carey, 2014). Further, evidence suggests that these casual sexual encounters or “hookups” are increasing at HEIs and that most committed romantic relationships at HEIs begin with a casual hookup (England et al., 2008; Garcia et al., 2012; Lambert et al., 2003). Research that solely focuses on sexual violence within dating or intimate partner relationships is limited as it fails to consider other encounters where sexual violence can and does occur (Duval et al., 2018). Given the nuance in young people’s relationships, there is a need to explore the unique dynamics of sexual violence in this population.

Dating violence or intimate partner violence is inclusive of physical and psychological violence, in addition to sexual violence. Sexual violence has distinct attributes when compared to these other forms of violence. According to some estimates, perpetration rates of physical and psychological aggression in intimate relationships are consistent between men and women (Duval et al., 2018); however, on average, women are more likely to be victims of sexual violence when compared to men and men are more likely to perpetrate sexual violence when compared to women (Black et al., 2016; WHO, 2017). The terms “men” and “women” are used in this research intentionally to signify gender identity as opposed to sex.

### Perpetration and Victimization

The most recent systematic review of sexual violence at HEIs in the U.S. context found that the average prevalence of sexual violence perpetration (broadly defined) was approximately 29%, highlighting the substantial prevalence of perpetration among HEI students (Anderson et al., 2019). Yet institutional culture at HEIs “often promotes a mission of helping women help themselves to prevent sexual violence” (Wooten & Mitchell, 2016, p. 3). This is evident through the increasing popularity of bystander education, self-defense trainings, walk-home services, and warnings to limit consumption of alcohol or drugs (Wooten & Mitchell, 2016). Overall, these services do not fundamentally change the root causes and factors that lead to sexual violence against women (Wooten & Mitchell, 2016). Therefore, this research intentionally focuses on risk factors for sexual violence perpetration at HEIs, rather than sexual violence victimization, with the intent of consolidating an evidence base that can be used to inform perpetration prevention initiatives at HEIs (Sutherland et al., 2014).

### Risk and Protective Factors: Existing Research

From an epidemiological perspective, there are a wide range of risk and protective factors related to sexual violence (Centers for Disease Control and Prevention [CDC], 2019). Risk factors are variables associated with a higher likelihood of an adverse outcome occurring. In the context of sexual violence at HEIs, risk factors could include drug or alcohol use, previous delinquent or risk-taking behaviors, sexual history, and hostile attitudes about women (CDC, 2019). Other hypothesized risk factors could include socioeconomic status, employment, familial relationships, racial and ethnic background, as well as misogynistic societal gender norms that place the rights, desires, and autonomy of men ahead of women (CDC, 2019). Organizational and environmental factors such as alcohol availability and social or athletic groups fostering hypermasculine norms could play a role in sexual violence perpetration (Moylan & Javorka, 2020). Students at HEIs may be living away from home for the first time, lacking many of the informal and formal controls they are accustomed to, and be engaging in regular social and leisure activities exposing them to greater risks (Fisher et al., 1998; McCluskey-Fawcett et al., 2001).

In contrast, protective factors for sexual violence perpetration, factors that reduce the likelihood of an individual perpetrating sexual violence, may include good mental health, strong connection to community or family, academic success, positive parental role-modeling on how to approach conflict, and empathetic personality traits (CDC, 2019).

### Prospective Data

Prospective data and longitudinal study designs are a requirement for establishing risk and protective factors (Murray et al., 2009). These study designs survey the same participants at least twice over a set time period allowing for both within and between individual comparison. They produce less biased and more confident findings about risk and protective factors when compared to cross-sectional study designs (Murray et al., 2009). Prospective data, unlike cross-sectional data, can prove that the risk or protective factor occurred before the outcome, a key condition for establishing causality (Hill, 1965; Setia, 2016).

Despite the strength of prospective data in measuring risk and protective factors, longitudinal study designs are challenging to implement. They require more time and resources than cross-sectional surveys and necessitate continued participation from survey respondents, which could lead to compromised sample sizes (Setia, 2016). Despite these limitations, it is worthwhile to specifically examine longitudinal studies to rigorously identify risk and protective factors for sexual violence perpetration at HEIs (Murray et al., 2009; Yakubovich et al., 2018).

### Theoretical Models

Three models described by Zinzow and Thompson (2015) are commonly used to explain the relationship between risk factors and sexual violence perpetration at HEIs. First, *the ecological theory of sexual aggression* provides a holistic approach to linking early childhood experiences, peer norms, and cultural belief systems to sexual violence perpetration (Malamuth, 2003; Zinzow & Thompson, 2015). This theory has been substantiated by studies involving HEI students (Hall & Barongan, 1997; Ouimette & Riggs, 1998; Thompson et al., 2011) that have found childhood adversity and peer support for forced sex to be correlated with the perpetration of sexually violent acts (Zinzow & Thompson, 2015). A limitation to this theory is that it does not also include situational dynamics to explain why an individual may perpetrate an act of sexual violence (Fisher & Sloan, 2013; Fisher et al., 1998; Henson & Stone, 1999).

Second, *the confluence model* of sexual aggression—the most commonly used model to theorize sexual violence perpetration at HEIs—emerged out of the ecological theory of sexual aggression but provides a more specific approach. The confluence model combines a variety of different risk factors into the following two constructs: hostile masculinity, a distrusting and angry disposition toward women, and impersonal sexual orientation, a desire to engage in uncommitted sexual relationships for physical gratification (Malamuth, 2003). Many studies at HEIs have shown that hostile masculinity, traditional attitudes toward gender roles and sexual relationships, and acceptance of rape myths are linked to sexual aggression (e.g., see Abbey, 2011; Abbey et al., 2001; DeGue & DiLillo, 2004). Sexual violence perpetrators have also been found to engage in impersonal or casual sex, have their first sexual experience at an earlier age, and have more consensual sexual partners (DeGue & DiLillo, 2004).

Third, *the reactance theory of sexual coercion*, which is similar to the confluence model, emphasizes how the refusal of sex by a desired sexual partner can act as a trigger for sexual violence perpetration, suggesting that individuals with narcissistic personality traits such as lack of empathy have a disposition to respond to sexual rejection with sexual violence (Zinzow & Thompson, 2015). The relationship between a lack of empathy and sexual violence perpetration has also been found in HEI settings (Mann & Hollin, 2007; Thompson et al., 2011).

Ultimately, perpetrators do not always share the same motivations or tactics for engaging in sexual violence. It is essential to measure a wide range of risk factors that emerge from different theories to understand how these factors interact with each other and an individual’s environment and experiences (WHO, 2003). Godenzi et al. (2001) put forth an *integrated gendered social bond and male peer support theory* to explain the phenomena of sexual violence at HEIs more comprehensively. Godenzi et al. (2001) claim that sexual violence is a result of “men’s attempt to maintain a social bond with a conventional or traditional social order marked by gender inequality” (p. 11). Group behaviors, attitudes, and ideologies can promote, justify, and legitimize sexual violence, and perpetration of sexual violence can be a learned behavior through social interactions with male peers (DeKeseredy & Schwartz, 2013). Peer groups in the HEI setting may promote rape culture, given the patriarchal institutional environment of HEIs (Godenzi et al., 2001). Specifically, male groups such as fraternities and sports teams have been found to foster a hypermasculine culture in which men feel pressure and/or entitlement to use coercion and force for sex (Godenzi et al., 2001). Testing the above theories through meta-analyses has the potential to inform the development and implementation of proactive and responsive interventions to reduce sexual violence at HEIs.

### Study Aims and Rationale

This review aims to contribute to three gaps in our understanding of the evidence on risk and protective factors for sexual violence perpetration at HEIs. Existing systematic reviews related to this topic have (1) not explored the risk and protective factors for sexual violence perpetration specifically in the HEI context (Yakubovich et al., 2018), (2) not examined sexual violence independently from intimate partner or dating violence (Duval et al., 2018), or (3) only focused on victimization (Greathouse et al., 2015). This study represents the first systematic, meta-analytic review of all prospectively measured risk and protective factors for sexual violence perpetration at HEIs.

## Method

### Inclusion and Exclusion Criteria

We developed a set of prespecified inclusion and exclusion criteria informed by related systematic reviews (Greathouse et al., 2015; Yakubovich et al., 2018). This was a global search; no limits were placed on the geographical location of included studies, although the search was conducted only in English and studies needed to be written in English to be included.

We included studies based on the sample and outcome (men’s perpetration of peer-to-peer sexual violence against women), setting (HEIs), and study design (quantitative, longitudinal studies assessing risk and/or protective factors of sexual violence). We only included longitudinal study designs to demonstrate that the potential risk factor preceded the outcome (Murray et al., 2009).

### Literature Search

We searched the following electronic bibliographic databases: MEDLINE, PsycINFO, EMBASE, and PsycARTICLES. These databases were selected based on searches from related systematic reviews (Duval et al., 2018; Greathouse et al., 2015; Yakubovich et al., 2018) as well as an initial inquiry into where prominent articles were contained. We also searched the following three databases for gray literature: United Nation Women’s Global database on violence against women, the Global Health Observatory Data Repository, and the WHO’s Institutional Repository for Information Sharing. We hand searched the reference lists of included articles to retrieve additional studies not already identified.

We conducted a preliminary literature review of the topic and related systematic reviews to develop a list of key words and synonyms of these key words to describe each component of the research question. These words informed the design of the final search string which balanced sensitivity and specificity. We adapted the search string for each database by adding appropriate subject heading terms. The search was initially conducted in April 2019 and again in November 2019 to check for newly published studies. Two reviewers screened titles and abstracts for eligibility at each stage. The agreement rate between researchers was 98.6%. We conducted full-text screening of articles that initially met the inclusion criteria to ensure eligibility.

### Screening, Coding Procedures, and Data Extraction

After completing the search, we imported all records into the reference manager *Rayyan* and screened studies using the prespecified inclusion and exclusion criteria. Data on study characteristics (sample, outcome type, outcome measure, prevalence of outcome, study size, time frame, and location) and effect estimates for each risk factor were extracted from each included study. The data from the last possible time point were extracted to establish a stronger relationship between the risk factor and the outcome. Data were extracted electronically using a spreadsheet to identify similarities between studies and to uncover gaps in the literature.

When possible, the last time point measured in each study was extracted for each meta-analysis to select the data at its greatest maturity. Further, if two or more degrees of sexual violence severity were reported, the most severe form of sexual violence was selected (e.g., “forcible rape” would be selected instead of “verbal pressure for unwanted contact”; White & Smith, 2004, p. 193).

### Quality of Studies: Risk of Bias Assessment

Risk of bias assessments evaluate the quality of risk and protective factors measured in included studies (Murray et al., 2009). The quality of these factors directly impacts the strength and relevance of the findings (Murray et al., 2009). The review team adapted the *Cambridge Quality Checklists* for systematic reviews to critically appraise the quality of the included studies, strengthen the claims made in the review, and assess the state of the research in the field (Murray et al., 2009). This tool has three checklists that build upon each other to evaluate the quality of the evidence for drawing conclusions on correlational and causal relationships (Table 1).

**Table 1.** Adapted Cambridge Quality Checklists.^a^

Correlate Score (Sum Out of 5)_	
Sampling method	
1	Total population sampling *or* random sampling
0	Convenience sampling
Response rates	
1	Response and retention rates ≥ 70%
0	Response rate < 70% *or* retention rate < 70% *or* not known
Sample size	
1	Sample size ≥ 400
0	Sample size < 400
Measure of risk or protective factor ^b^	
1	Reliability coefficient ≥ .75 *and* reasonable face validity
	*or* criterion or convergent validity coefficient ≥ .3
	*or* more than one instrument or information source used to assess correlate
0	None of the above or not known
Measure of outcome	
1	Validated scale
	*or* reliability coefficient ≥ .75 *and* reasonable face validity
	*or* more than one instrument *or* information source used to assess outcome
0	None of the above or not known
Risk/protective factor score (out of 3)_	
1	Cross-sectional data
2	Retrospective data
3	Prospective data
Causal risk factor score (out of 7)_	
1	Study without a comparison group
	No analysis of change
2	Inadequately controlled study
	No analysis of change
3	Study without a comparison group
	With analysis of change
4	Inadequately controlled study
	With analysis of change
5	Controlled nonexperimental study
	No analysis of change
6	Controlled nonexperimental study
	With analysis of change
7	Randomized experiment
	Targeting a risk/protective factor

^a^ This version of the Cambridge Quality Checklists (Murray et al., 2009) is based on the version used in a related review on risk and protective factors for intimate partner violence conducted by Yakubovich et al. (2018).

^b^ The measure used for each risk/protective factor assessed in a study was appraised separately. The ratings provided in the table that includes the scores are average of the ratings for each risk and protective factor.

## Analysis

### Computation of Effect Sizes

In most studies included in the review, the outcome was measured dichotomously (participants either perpetrated a certain type of sexual violence or they did not or participants belonged to a certain trajectory group of sexual violence perpetration and not another group). Odds ratios (*OR*s) were used to assess the relationship between a risk factor and sexual violence. When *OR*s were not used and outcomes were measured as continuous variables, we transformed means and standard deviations or effect sizes from *F* tests or *t* tests to *OR*s using David Wilson’s Online Effect Size Calculator from the Campbell Collaboration (Wilson, n.d., https://campbellcollaboration.org/escalc/html/EffectSizeCalculator-Home.php, Retrieved July 20, 2019). Prior to completing the meta-analyses, we converted *OR*s into log odds. We calculated variance (*s*^2^) using the standard error (*SE*; Colditz et al., 1994). We converted confidence intervals (CIs) of a reported *OR* to *SE* when *SE* was not reported (Colditz et al., 1994).

### Meta-Analysis

We summarized the quantitative results from each included study in a table and conducted the meta-analyses using R software (version 3.5.1). Given the methodological heterogeneity between studies, we conducted simple random effects (RE) meta-analyses using the DerSimonian and Laird method (the method-of-moments estimator) to estimate the overall effects of different risk factors that were comparably assessed in more than one study (Dersimonian & Laird, 2015; Tipton, 2015). This is a standard method for RE meta-analyses, which accounts for within- and between-study variance in the study weights. In particular, studies are weighted by the inverse of the *SE* of the study-specific effect estimate and adjusted to account for between-study variance. We recorded heterogeneity, represented by *I*^2^, a statistic that measures the consistency between the effect sizes in each study, and τ^2^, an estimate of the between-study variance (Higgins & Green, 2008, the Cochrane Handbook for Systematic Reviews of Interventions section 9.5.2 and 9.5.4). We excluded two studies that used a daily diary approach for administering surveys (Testa et al., 2015, 2018). The daily diary methodological approach was deemed too different to compare findings with the other included studies; first, the daily diary studies asked about events that occurred in the past 24 hr when all other included studies asked about events in the past academic year, second daily diary studies took place over 56 days while all other included studies ranged from 1 to 4 years in duration.

### Quality Appraisal

We used the Grading of Recommendations, Assessment, Development, and Evaluations (GRADE) framework to summarize the quality of evidence for each risk factor (Guyatt et al., 2011). Four levels of GRADE evidence (very low, low, moderate, and high) assess the degree of similarity between the estimated effect and the true effect (Guyatt et al., 2008).

## Results

### Overview of Study Set

**Figure 1.**
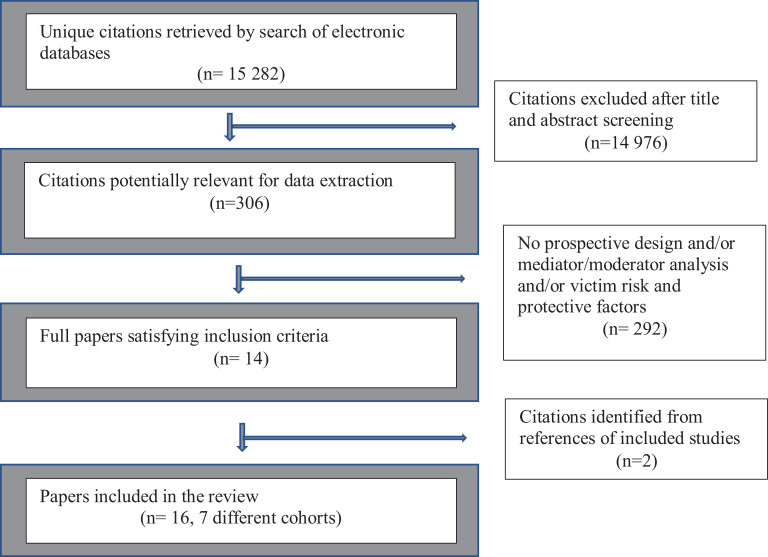
Flow diagram.

We screened 14,229 titles and abstracts and deemed 302 citations eligible for full-text review. After full-text screening, we included 14 articles. Hand searching the reference lists of the included articles yielded two more relevant articles. As a result, 16 articles across seven different samples were included in this study. The flow diagram (Figure 1) details the results of the screening process (Moher et al., 2009). All of the 16 included studies used data collected from students attending HEIs in the United States, and each study measured the outcome variable using self-reported perpetration. Studies were published between 2003 and 2019, and sample sizes ranged from 197 to 851 men. Each study reported on an average of eight risk and protective factors. The majority of factors studied were risk factors rather than protective factors.

### Risk of Bias Assessment

Overall, the risk of bias in all included studies is varied. Most studies used reliable measures for assessing risk factors (13 of 16 studies) and for assessing outcomes (14 of 16 studies). Thirteen studies used a version of a validated tool developed by Koss and colleagues (2007) called the *Sexual Experiences Survey (SES).* The *SES* asks participants to report on the frequency with which they have engaged in a list of behaviors of increasing severity from “I stared at someone in a sexual way or looked at the sexual parts of their body after they had asked me to stop” to “I put my penis or I put my fingers or objects into a woman’s vagina without her consent” (Koss et al., 2007). The most common reasons for low quality of correlational evidence can be attributed to inadequate sampling methods (*n* = 9), small sample sizes (*n* = 8), and poor response rates (*n* = 10).

Of the studies assessed, nine used inadequate sampling methods and relied on convenience sampling rather than whole population or random sampling. For example, Abbey and McAuslan (2004) recruited volunteer samples through advertisements in student newspapers, fliers, and classroom announcements (Abbey et al., 2001, p. 789). As findings from studies that used convenience or nonrandomized samples have limited external validity, total population sampling approach is the standard for studies on risk factors (Murray et al., 2009). Seven articles (from three distinct samples) attempted to take a total population approach by emailing all eligible students attending the HEI under study (Testa & Cleveland, 2017; Thompson et al., 2015; Thompson & Morrison, 2013; Thompson et al., 2013; White & Smith, 2004, 2009; Zinzow & Thompson, 2015). Many studies reported small sample sizes (*n* = 8) and poor retention rates (*n* = 10). This is likely because many of the studies used multiple waves of follow-up assessments, with the last wave being up to 4 years after the initial survey.

We assessed studies to determine whether the study design supported the evaluation of causal risk factors (as opposed to correlates; Murray et al., 2009). The Cambridge Quality Checklists criteria for establishing causality is as follows: Risk factors must be measured using longitudinal methods rather than retrospective or cross-sectional designs; risk factors should be randomized if possible; studies must include an analysis of change; and studies must control for confounding variables prior to the assessment of a risk factor (Murray et al., 2009).

Due to our selection criteria, all included studies used a longitudinal design and as a result received full points for the Risk Factor Score on the Risk of Bias Assessment. This rigorous inclusion allowed us to control for an aspect of study quality. We included “high-quality, nonrandomized” studies as the top-rated criterion, given that randomization was not possible in the included studies (Murray et al., 2009, p. 12). All of the studies in this review were designed to assess an analysis of change, except Thompson and Morrison (2013). We took a liberal definition of analysis of change to include between-subject variation rather than just within-subject variation which is consistent with the approach taken by Yakubovich et al. (2018) in a related systematic review. Finally, all but one study (Loh, 2003) controlled for confounding variables, but there was little consistency on which control variables were used across studies. Examples of covariates include social desirability (Abbey & McAuslan, 2004), previous perpetration (Zinzow & Thompson, 2015), and relationship status (Walters, 2019).

### Meta-Analyses

**Figure 2.**
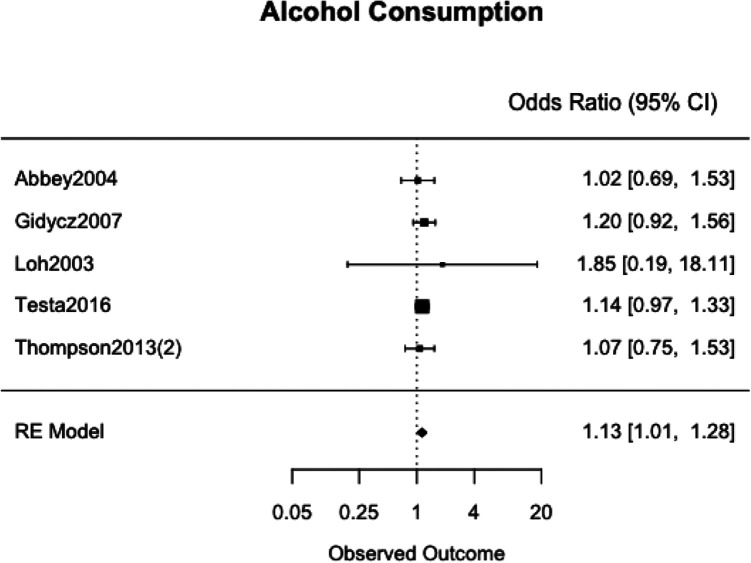
Alcohol consumption.

**Figure 3.**
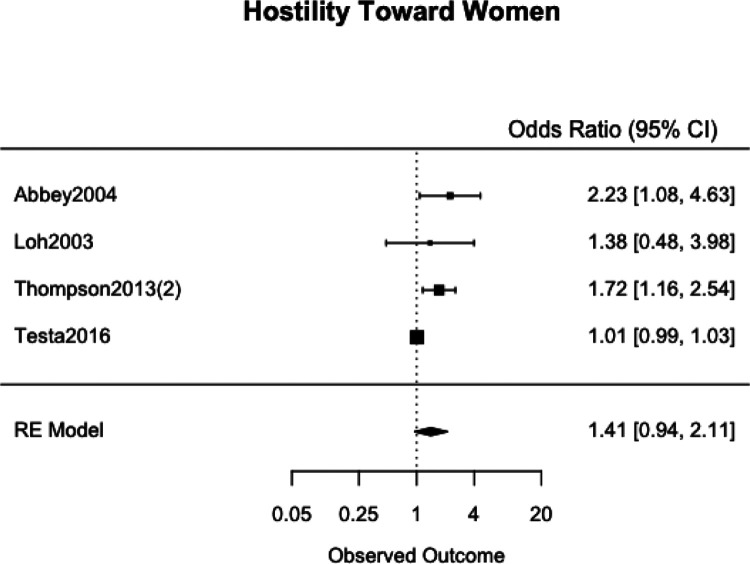
Hostility toward women.

**Figure 4.**
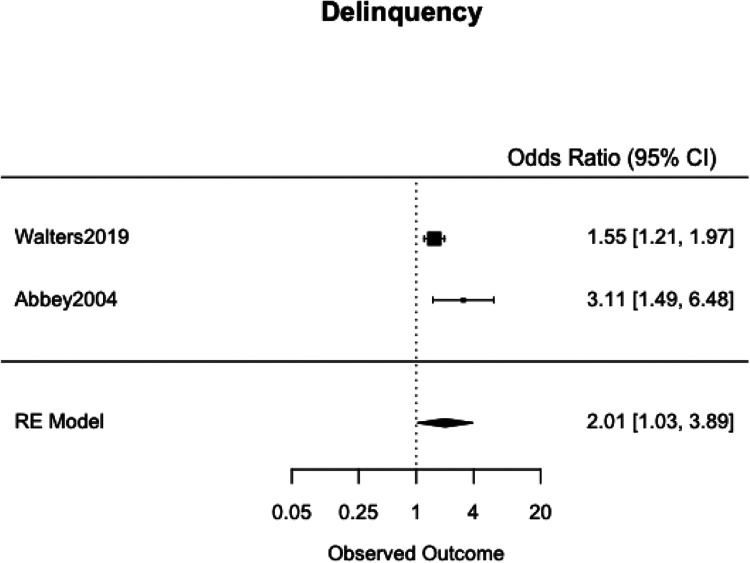
Delinquency.

**Figure 5.**
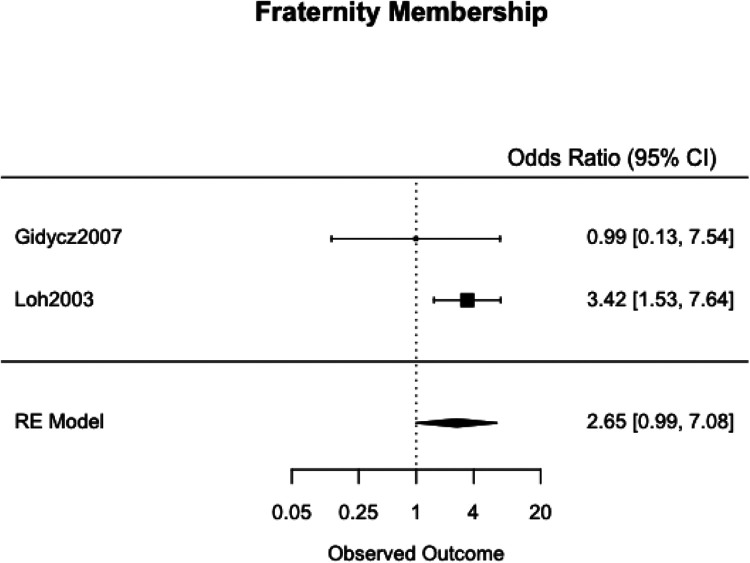
Fraternity membership.

**Figure 6.**
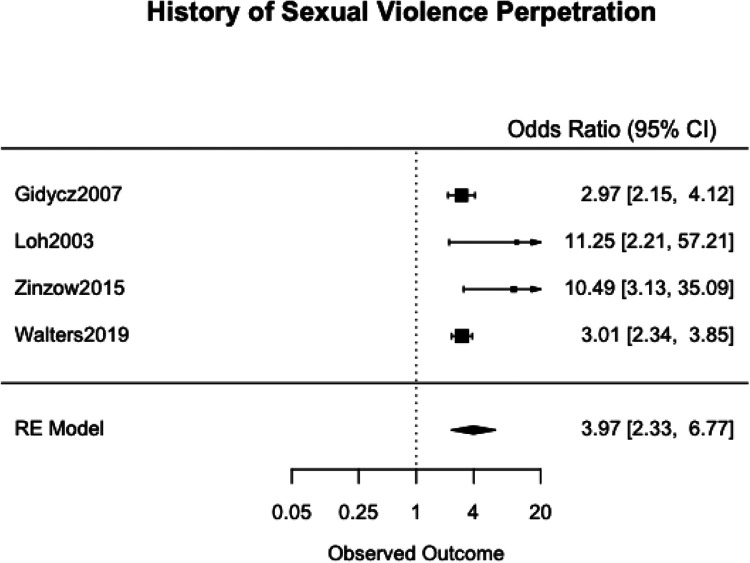
History of sexual violence perpetration.

**Figure 7.**
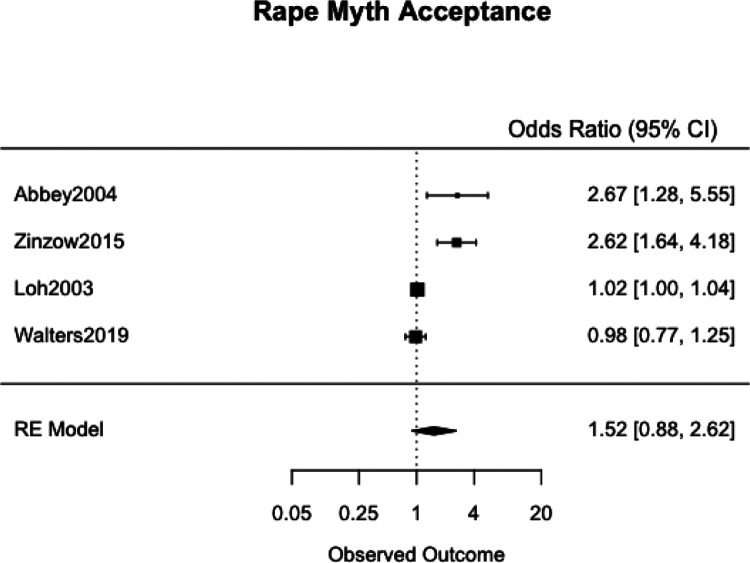
Rape myth acceptance.

**Figure 8.**
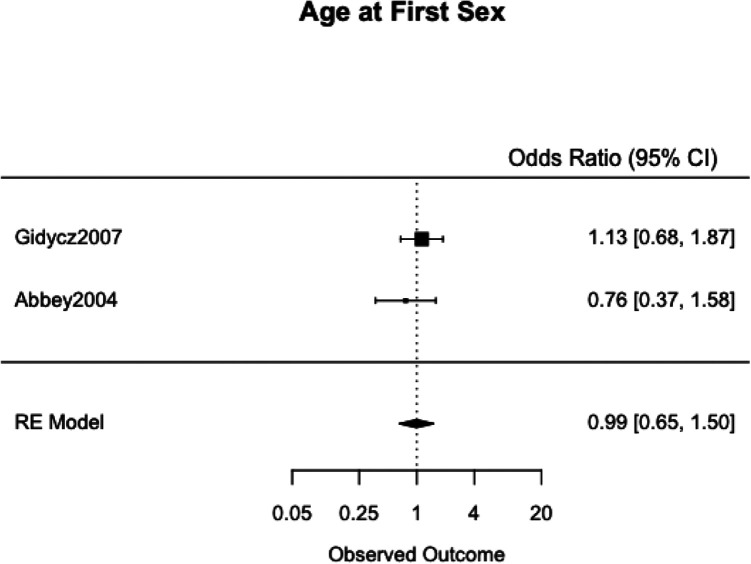
Age at first sex.

**Figure 9.**
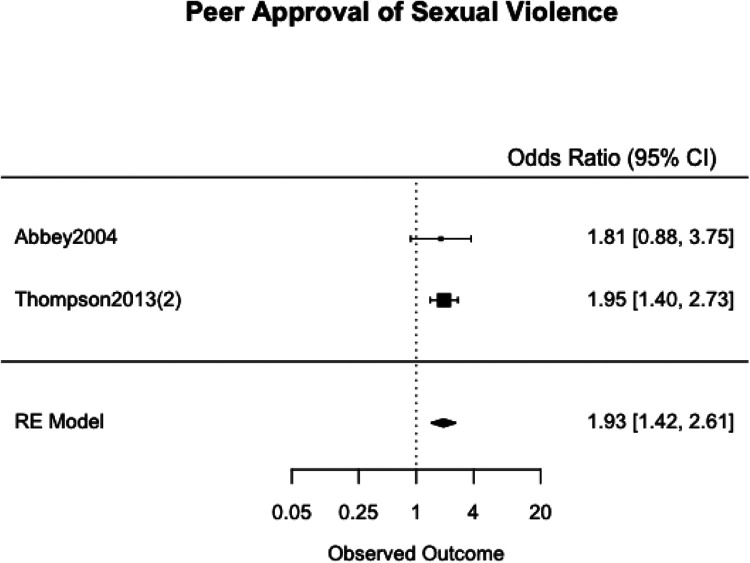
Peer approval of sexual violence.

We meta-analyzed the effect estimates for eight different risk factors from six different samples using a simple RE model. Meta-analysis was not possible in many cases as studies reported on different risk and protective factors. The forest plots for each meta-analysis are provided in Figures 2–9. Each forest plot corresponds to a risk factor and provides the *OR* and CIs from each study. The diamond at the bottom of each figure represents the pooled *OR* for a specific risk factor. Table 2 includes a summary of the key findings including results for the meta-analyses and the quality of the evidence assessment (GRADE).

**Table 2.** Summary of Results.

Variables	*N* men in Meta-analysis	*N* studies in Meta-analysis	Model Estimate	*OR* [95% CI]	*p* Value	Heterogeneity *I*^2^ (τ^2^)	Quality of the Evidence (GRADE)
Alcohol consumption	2,554	5	0.1252	1.13 [1.01, 1.28]	.048	0.0% (0.00)	⊕⊕⊕⊕ HIGH
Hostility toward women	2,218	4	0.3420	1.41 [0.94, 2.11]	.0959	70.55% (0.1010)	⊕⊕⊕ˆ MODERATE
Delinquency	1,048	2	0.6960	2.01 [1.03, 3.89]	.0396	68.21% (0.1669)	⊕⊕⊕ˆ MODERATE
Fraternity membership	570	2	0.9749	2.65 [0.99, 7.08]	.0518	19.29% (0.1483)	⊕⊕⊕ˆ MODERATE
History of perpetration	1,894	4	1.34	3.8 [1.70, 8.58]	.0012	89.83% (0.5033)	⊕⊕⊕ˆ MODERATE
Rape myth acceptance	1,755	4	0.1312	1.52 [0.88, 2.62]	.1312	93.96% (0.2628)	⊕⊕⊕⊕ HIGH
Age at first sex	533	2	−0.0077	0.99 [0.65,1.50]	.9710	0.0% (0.00)	⊕⊕⊕ˆ MODERATE
Peer approval of sexual violence	992	2	0.6569	1.93 [1.42, 2.61]	<.0001	0.0% (0.00)	⊕⊕⊕⊕ HIGH

#### Alcohol consumption

We used five studies with a combined 2,554 participants to meta-analyze alcohol consumption as a risk factor. Two studies, Gidycz et al. (2007) and Loh (2003), used The Drinking and Drug Habits Questionnaire to assess alcohol use. Gidycz et al. (2007) adapted their version from Collins et al. (1985), while Loh (2003) relied on their version of Cahalan et al. (1969) and included a Volume Variability Index developed by Cahalan and Cisin (1968) to measure alcohol use. Two studies asked questions about the drinking behavior of participants over the past 30 days (Abbey & McAuslan, 2004; Thompson et al., 2013). Testa and Cleveland (2017) measured alcohol consumption by asking about the amount of times a participant drank “five or more drinks in a single occasion during the past semester” (Testa & Cleveland, 2017, p. 7). The Testa and Cleveland (2017) study was the only study that studied this risk factor without providing any information on the reliability or validity of the measure. Across all studies, a one-unit increase in men’s alcohol consumption was associated with 13% higher odds of perpetrating sexual violence, 95% CI [1%, 28%], with no heterogeneity between studies (*I*^2^ = 0.0%, τ^2^ = .00; Figure 2).

#### Hostility toward women

We used four studies with a combined 2,218 participants to meta-analyze hostility toward women as a risk factor. Three studies used validated measures: Loh (2003) used the Hypergender Ideology Scale (Hamburger et al., 1996), Thompson et al. (2013) used an adapted Hostility Toward Women Scale, and Testa and Cleveland (2017) used a scale adapted from Lonsway and Fitzgerald (1995) to assess hostility toward women. Abbey and McAuslan (2004) measured “callous attitudes toward women” by coding responses based on the extent to which they reflected callous attitudes toward women—for example, describing women in disparaging terms such as “sexual commodities, or solely in terms of their physical appearance” (Abbey & McAuslan, 2004, p. 7). Hostile attitudes toward women acted as a risk factor for sexual violence in some settings. The pooled effect showed a one-point increase in men’s average hostility toward women score to be associated with a 41% increase in likelihood of perpetration, 95% CI [6%, 111%] (Figure 3). The high heterogeneity (*I*^2^ = 70.55%, τ^2^ = .1010) in the meta-analysis is based on one study (Loh, 2003), which limits the strength of this relationship.

#### Prior delinquency

We used two studies with a combined 1,048 participants to meta-analyze prior delinquency as a risk factor. Walters (2019) did not use a reliable measure and assessed general offending using the self-reported number of times respondents engaged in six distinct “non-sexual offenses over the past year” (p. 1315). Abbey and McAuslan (2004) tested for delinquency using a validated 13-item delinquency scale (with a six-point response scale; p. 8). Prior delinquency emerged as a risk factor, associated with 101% higher odds of sexual violence perpetration, 95% CI [3%, 289%] (Figure 4). This result is contextualized by the high heterogeneity between the studies (*I*^2^ = 68.21%, τ^2^ = .1669) which could in part be due to the differing measures used in each study.

#### Fraternity membership

We used two studies with a combined 570 participants to meta-analyze fraternity membership as a risk factor. Both Gidycz et al. (2007) and Loh (2003) determined fraternity membership using a yes or no question in demographic questionnaires. Gidycz et al. (2007) reported on data collected 3 months after the baseline survey, and Loh (2003) reported data from both a 3-month follow-up and a 7-month follow-up. We chose to use the 3-month follow-up data to ensure consistency. Fraternity membership was associated with a 165%, 95% CI [99%, 608%], increased likelihood of perpetrating sexual violence with moderate heterogeneity (*I*^2^ = 19.29%, τ^2^ = .1483; Figure 5).

#### History of sexual violence perpetration

We used four studies with a combined 1,894 participants to meta-analyze history of sexual violence perpetration as a risk factor. Three studies used the *Sexual Experiences Survey* developed by Koss and Gidycz (1985) to assess for perpetration, Gidycz et al. (2007) and Walters (2019) defined history of sexual violence perpetration to be perpetration that occurred between the age of 14 and the start of their surveys, and Zinzow and Thompson (2015) and Loh (2003) assessed for any perpetration prior to attending the HEI. History of sexual violence was associated with an increase of risk of sexual violence perpetration by 297%, 95% CI [133%, 577%], with high heterogeneity (*I*^2^ = 89.83%, τ^2^ = .5033; Figure 6).

#### Rape myth acceptance

We used four studies with a combined 1,755 participants to meta-analyze rape myth acceptance as a risk factor. Abbey and McAuslan (2004) used validated measures of rape supportive attitudes to assess “rape myth acceptance, adversarial sexual beliefs, and acceptance of interpersonal violence” (p. 7). Zinzow and Thompson (2015) assessed for rape supportive beliefs and norms using a reliable 19-item *Rape Supportive Beliefs Scale* (Lonsway & Fitzgerald, 1995), while Loh (2003) used the *Illinois Rape Myth Acceptance Scale*. Walters (2019) was the only study to not report using a validated or reliable measure and instead evaluated “blame attributions” which was defined as “the tendency to blame the female victim of a sexual assault” (p. 1315). The pooled effect showed a one-point increase in men’s average rape myth acceptance score to be associated with a 52% increase in odds of sexual violence perpetration, but the association was variable, 95% CI [12%, 162%], with high heterogeneity (*I*^2^ = 93.96%, τ^2^ = .2628; Figure 7). This demonstrates differences in the strength and predictive power of rape myth acceptance as a risk factor across studies, which could, for instance, be due to the different measures used across studies and the years in which the surveys were conducted.

#### Age at first sex

We used two studies, with a combined 533 participants, to meta-analyze the risk factor of age at first sex. Both Abbey and McAuslan (2004) and Gidycz et al. (2007) asked at what age participants first had consensual sexual intercourse in demographic questionnaires. We found no evidence of a meaningful association between age at first sex and sexual violence perpetration (pooled *OR* = 1.01, 95% CI [1.35, 1.50]) and no heterogeneity between the studies (*I*^2^ = 0.00%, τ^2^ = .00; Figure 8).

#### Peer approval of sexual violence

We used two studies with a combined 992 participants to meta-analyze peer approval of sexual violence as a risk factor. Abbey and McAuslan (2004) used a five-point scale to ask six questions that assessed the extent to which participants’ friends would “approve of using various strategies to obtain sex with a woman, including lying to her, forcing her, and getting her drunk” (p. 8). Thompson et al. (2013) also asked respondents six questions regarding “their perceptions of their current set of friends approval and pressure for engaging in various coercive strategies to have sex with women” (p. 6). A one-point increase in men’s average peer approval of sexual violence score increased the odds of sexual violence perpetration by 93%, 95% CI [42%, 161%], with no heterogeneity across study estimates (*I*^2^ = 0.00%, τ^2^ = .00; Figure 9).

## Discussion

The aim of this study was to gather and synthesize the available evidence on risk and protective factors for male sexual violence perpetration at HEIs in order to develop an understanding of what is needed for effective interventions. Key strengths of this study are the quality of evidence for risk factors (as determined by the specified inclusion and exclusion criteria) used to conduct meta-analyses and to draw conclusions and the systematic processes of the review which allowed us to determine the extent of the evidence for risk and protective factors for sexual violence at HEIs.

History of perpetration emerged as the strongest predictor of sexual violence perpetration at HEIs. Existing theories for sexual violence often focus on the HEI environment itself as contributing to a culture where sexual violence can occur (Fisher, 2000). Our findings do not discount the saliency and relevance of other risk factors involving the HEI environment; however, they draw attention to the importance of addressing the issue of sexual violence among students prior to them attending an HEI.

Alcohol consumption was associated with increased odds of sexual violence perpetration; however, the effect size (14%) was smaller than anticipated, given the hypothesized role of alcohol in sexual violence perpetration at HEIs (Abbey, 2011; Abbey et al., 2014). This finding could reflect the types of measurement tools used in the included studies; studies measured general alcohol consumption by individuals rather than alcohol consumption during an incident of sexual violence.

Despite substantial prior claims highlighting their importance, hostile masculinity and men’s rape myth acceptance did not strongly predict sexual violence perpetration across included studies (Abbey, 2011; DeGue & DiLillo, 2004). This contradicts the confluence model of sexual aggression linking personal beliefs of hostile masculinity and rape myths to sexual violence perpetration. Fraternity membership and peer approval of sexual violence were found to be more decisive predictors of sexual violence perpetration, with little heterogeneity, but the strength of this relationship could diminish if more studies were available to be included. While more research is needed to increase the volume of studies available to meta-analyze and substantiate these preliminary findings, our results indicate the relative importance of peer groups in influencing behavior among HEI students when compared to individual beliefs. This finding aligns with the gendered social bond and male peer support theory used to explain sexual violence (Godenzi et al., 2001).

One interpretation of these findings is that even if individual HEI students hold beliefs that perpetuate rape culture, fear of being excluded from peer groups who exhibit opposing beliefs could mitigate sexually violent actions from occurring in some settings. Conversely, individual HEI students could be placed at a higher risk of perpetrating sexual violence if their peer groups (in this case fraternities) condone this behavior or if their peers approve of sexual violence.

This finding substantiates the ecological theory of sexual aggression, which highlights the relationship between peer norms and sexual violence perpetration. It also draws attention to the importance of the gendered social bond and male peer support theory in providing an integrated theoretical framework combining the individual, relational, and contextual risk factors for sexual violence as well as the ways in which they interact. For example, fraternity membership and interest in fraternity membership have been linked to alcohol consumption, rape myth acceptance, and greater proclivity to perpetrate sexual violence (Seabrook et al., 2018). This interplay between fraternity membership, alcohol consumption, peer approval, and rape myth acceptance showcases how risk factors for sexual violence must be understood in relation to each other in order to develop effective interventions.

Finally, the age at which an individual first had sex did not have a meaningful relationship with sexual violence perpetration. This opposes findings from studies of cross-sectional survey data by Senn et al. (2000), Abbey et al. (1998), and Koss and Dinero (1988), which show that men who were sexually active at an earlier age were more likely to perpetrate sexual violence when compared to men who were not. The absence of an association, however, is not evidence of no association. A variety of confounding relationships between risk factors could diminish the magnitude of each factor’s individual predictive power.

### Risk Factors

A wide range of risk factors were included in this study. Given that the methodology did not prespecify a set of risk and protective factors to review, a key aim of this study was to explore what types of risk and protective factors have previously been assessed. Alcohol consumption was the most common risk factor assessed in the included articles (*n* = 9). Excluding alcohol consumption, very few studies reported on similar factors, which impacted the number of studies included in each meta-analysis. More studies that measure commonly hypothesized risk factors for perpetration such as history of victimization and group membership (e.g., fraternity and sports teams) would be useful for future research.

Even though this study searched for articles that investigated any risk or protective factor, no study explicitly discussed protective factors and no study measured the same protective factor as another study. Protective factors are not as consistently defined and measured when compared to risk factors (Farrington & Ttofi, 2011). Further theorizing on what reduces the likelihood that of HEI students perpetrating sexual violence and future research that prospectively measures the relationship between these protective factors and the outcome of sexual violence perpetration is needed. A good understanding of protective factors is valuable for developing strength-based models of prevention and response rather than solely relying on deficit-based models.

### Quality of Evidence

The overall risk of bias in the included studies is varied, and the quality of the evidence included in this study is moderate to high. The most common reasons for bias included inadequate sampling methods, sample sizes, and response rates. Convenience sampling in studies could lead to bias in participation. Sample size and response rates were likely low due to the prospective nature of these studies, which requires more time and investment from participants. The risk of bias assessment contextualizes the findings of the meta-analysis and draws attention to the strengths and limitations of longitudinal study designs on sexual violence perpetration at HEIs. Our strict inclusion and exclusion criteria that resulted in methodologically strong longitudinal studies allow us to be confident in our findings.

Every risk factor that was meta-analyzed was given a GRADE rating of moderate to high. Typically, high GRADE ratings are reserved for randomized control trials; however, several studies were able to increase their GRADE rating due to their large magnitude of effect size (Guyatt et al., 2008). A key factor limiting the quality of evidence of some included studies which was not accounted for in the risk of bias assessment or the GRADE assessment was the large number of factors measured in each study (Thompson & Morrison, 2013, *n* = 17; Abbey & McAuslan, 2004, *n* = 15; Loh, 2003, *n* = 46). It is problematic to try to find relationships between a large number of factors in one study (even if they were prespecified) as this can lead to “p-hacking,” cherry-picking, multiplicity (multiple variables to measure the same factor), and generating hypotheses after the results, in attempts to find statistically significant findings (Goodman et al., 2016). Replication is important for determining whether findings in studies testing a large number of factors are reproducible and reliable.

### Heterogeneity

Four of the eight meta-analyses had high heterogeneity. The meta-analysis for history of perpetration had high heterogeneity as it showed substantial variation in the magnitude of the effect. However, all of the individual study effect sizes were found to be significant predictors in both their direction and magnitude. Heterogeneity in this case does not raise any doubts about the importance and significance of the risk factor, but there is ambiguity surrounding exactly how large the effect size is. The high heterogeneity in the case of rape myth acceptance and hostile attitudes toward women contributed to the inconsistent pooled estimates. Heterogeneity in these cases is likely a result of the diversity of measurement tools used between studies to assess these risk factors: For example, heterogeneity within the meta-analysis may have been reduced if studies used the same validated tool to measure rape myth acceptance.

## Limitations

### Limitations of This Review

First, we did not investigate mediator and moderator analyses. Searching for and including this methodological design would have increased the number of studies synthesized in the review and provided insight into the relationship between interacting risk factors, potentially enhancing the nuance and applicability of the findings. Second, the scope of this research did not include sexual violence within the LGBTQ+ community. We made this decision to guide intervention directions for men’s perpetration of sexual violence against women as the most common type of sexual violence in HEIs, which has also been the predominant focus of primary etiological research (Black et al., 2016). The inclusion of sexual violence among gender and sexual minorities may have yielded different results, and it is important for future primary and secondary research to explore these differences. Third, this review only searched for studies in English and only included studies in English. Lastly, this review did not fully explore the confounders accounted for in studies or synthesize findings that could not be meta-analyzed.

### Limitations of the Evidence Base

All of the available studies drew upon samples of students attending HEIs in the United States. This restricts the generalizability of our findings and demonstrates the need for primary longitudinal research on sexual violence in HEIs from outside the United States. There was also a small number of studies eligible to meta-analyze for each risk factor. This is the result of a limited evidence base that has mainly focused on victimization rather than perpetration and relies upon cross-sectional survey designs rather than longitudinal approaches. A consequence of meta-analyzing a small number of studies is the high heterogeneity present in four of the eight risk factors assessed. In focusing on longitudinal designs and perpetration, this review demonstrates these limitations of the existing evidence base, which is an additional contribution to the literature.

There is also an overreliance on individual as opposed to structural-level risk factors measured in longitudinal study designs. This study was only able to meta-analyze individual-level risk factors. Structural risk factors for sexual violence are often more challenging to isolate and measure, but they are useful for understanding the contextual factors that interact with individual risk factors and perpetuate sexual violence (Blankenship et al., 2000).

## Future Research Recommendations

Future research should address the limitations in the existing evidence base. First, there is a need for rigorous research in countries other than the United States. Second, although men’s violence against women takes up the majority of the burden of sexual violence, gender minorities face disproportionate rates which should be explored in future research. Third, research should begin to theorize and measure protective factors to determine the characteristics of men who do not perpetrate sexual violence so that HEIs can promote these characteristics through programming. Fourth, studies could benefit by disaggregating outcome data in their analyses based on new and repeat perpetrators in the HEI setting and by the level of severity of the violence. Fifth, future reviews on risk factors for sexual violence at HEIs should synthesize mediators and moderator studies and examine the interactions between risk and protective factors. Sixth, more replication studies are needed to substantiate the risk factor associations identified in this review. Greater consistency between studies on risk and protective factors, especially in terms of measurement, will allow for stronger conclusions. Lastly, future longitudinal research could measure the role that policies and systems embedded within HEIs have in influencing norms around gender and violence, perceptions of accountability, and alcohol use that can perpetuate sexual violence. An exploration of the structural contexts that make HEI environments more or less conducive to perpetuating or preventing sexual violence is essential.

## Conclusion

While sexual violence at HEIs is a well-established phenomenon, this is the first study to systematically review and meta-analyze prospective data on the risk factors of male perpetration of sexual violence at HEIs. The findings can be used by HEIs (especially those based in the United States) to inform future research and to bolster training, awareness campaigns, and prevention initiatives for past and potential perpetrators (see “Implications” section).

History of sexual violence perpetration prior to attending an HEI was found to be the strongest predictor for sexual violence perpetration at an HEI, which suggests that sexual violence prevention programming needs to occur before students enter HEIs. Prevention programs could be instated at the high school and even elementary school levels. At the elementary school level, programming on age-appropriate bystander behavior and healthy relationships that challenge traditional gender norms and foster the importance of respecting the choices and bodies of peers could set the foundation for sustained change in reducing sexual violence at HEIs. Further, HEIs could implement strategies for targeting students who have previously perpetrated sexual violence.

The finding that peers play a role in determining whether an individual will perpetrate sexual violence provides evidence for the continued implementation of bystander programming at HEIs, as well as sexual violence prevention programming that targets norms and culture at HEIs as well as fraternities, and sports teams.

### Implications

#### Practice

- Widespread implementation of bystander intervention programming at HEIs.
- HEIs mandate (or to enhance, if already in place) training for peer groups such as fraternities and sports teams on sexual violence and respond to sexual violence perpetrated within these groups appropriately.

#### Policy

- Governments encourage education systems to include consent and sexual health training in high schools and elementary schools.

#### Research

- Theorize and conduct studies on protective factors for sexual violence perpetration at HEIs.
- Conduct more prospective and longitudinal research on risk and protective factors of sexual violence at HEIs outside of the U.S. context.

## Supplemental Material

Supplemental Material, sj-pdf-1-tva-10.1177_1524838020970900 - Risk and Protective Factors for Men’s Sexual Violence Against Women at Higher Education Institutions: A Systematic and Meta-Analytic Review of the Longitudinal Evidence Click here for additional data file. Supplemental Material, sj-pdf-1-tva-10.1177_1524838020970900 for Risk and Protective Factors for Men’s Sexual Violence Against Women at Higher Education Institutions: A Systematic and Meta-Analytic Review of the Longitudinal Evidence by Bridget Steele, Mackenzie Martin, Alexa Yakubovich, David K. Humphreys and Elizabeth Nye in Trauma, Violence, & Abuse
