# Supplementary material for: Risk and Protective Factors for Men’s Sexual Violence Against Women at Higher Education Institutions: A Systematic and Meta-Analytic Review of the Longitudinal Evidence
Source: Trauma Violence Abuse. 2020 Nov 11;23(3):716–32. doi: 10.1177/1524838020970900 (PMC9210109; doi:10.1177/1524838020970900)
Supplement: Supplemental Material, sj-pdf-1-tva-10.1177_1524838020970900 - Risk and Protective Factors for Men’s Sexual Violence Against Women at Higher Education Institutions: A Systematic and Meta-Analytic Review of the Longitudinal Evidence [file sj-pdf-1-tva-10.1177_1524838020970900.pdf]

Supplementary Materials

Search String

(student* or young* or undergraduate* or postsecondary or college* or universit* or school* or education*).ti. AND (risk or protect* or predict* or longitudinal or cohort or associat* or correlate* or contribute* or epidemiolog* or relat* or pathway*).tw. AND (sex* or assault* or aggress* or harass* or violen* or abus* or consen* or rape or forced sex or perpetrat* or victim*).ti. AND (class?mate* or peer* or acquaintance* or date* or dating or partner or intimate or relationship* or hookup* or friend* or boyfriend or girlfriend).tw.

Risk of Bias Assessment

Study ID	Date	Author	Sampling method	Response rates	Sample size	Measure of risk factor	Measure of outcome	Correlate score /5	Risk factor score /3	Causal score /7	Total score /15
1	2004	Abbey and McAuslan	0	0	0	1	1	2	3	6	11
2	2007	Gidycz et al.	0	1	0	1	1	3	3	6	12
2	2011	Gidycz et al.	0	1	0	0	1	2	3	4	9
3	2003	Loh	0	0	0	1	1	2	3	4	9
3	2005	Loh et al.	0	0	0	1	1	2	3	6	11
6	2010	Swartout and White	0	0	0	0	1	1	3	6	10
4	2016	Testa and Cleveland	1	0	1	1	1	4	3	6	13
7	2018	Testa et al.	0	0	0	1	0	1	3	6	10
7	2015	Testa et al.	0	0	0	1	0	1	3	6	10
5	2013	Thompson and Morrison(a)	1	1	1	1	1	5	3	5	13
5	2015	Thompson et al.	1	1	1	1	1	5	3	4	12
5	2013	Thompson et al. (b)	1	1	1	1	1	5	3	6	14
6	2019	Walters	0	0	1	1	0	3	3	6	11
6	2009	White and Smith	1	0	1	1	1	4	3	6	13
5	2015	Zinzow and Thompson,	1	1	1	1	1	5	3	6	14
6	2004	White and Smith	1	0	1	1	1	4	3	6	13

Description of Included Studies

Authors	Year	Study size	Outcome: Sexual violence type	Measure	Prevalence /incidence in population	Time frame	Risk and protective factors assessed
**Sample* 1**							
Abbey and McAuslan	2004	197	Sexual assault perpetration	Revised Sexual Experiences Survey, adapted to include 12 items (Koss et al., 1987)	14.2% (Incidence)	Last year	*Individual*:<br>Hostile gender-role beliefs<br>Callous attitudes toward women<br>Acceptance of verbal pressure<br>Frequency of heavy drinking<br>Delinquency<br>*Relational*:<br>Age at first date (years)<br>No. of dating partners and consensual sexual partners<br>Age at first consensual sex (years)<br>Typical drinking when dating<br>Typical drinking prior to consensual sex<br>No. of women misperceived<br>*Community/Societal*:<br>Peer approval of forced sex
**Sample 2**							
Gidycz, Warkentin, and Orchowski	2007	336	Sexual aggression	Sexual Experiences Survey, 10 item version (Koss & Oros, 1982)	10.2% (Incidence)	3 months ago	*Individual*:<br>History of sexual aggression<br>Average daily alcohol use<br>Problem drinking<br>History of verbal/physical aggression, and physical victimization<br>*Relational*:<br>Age of first sexual intercourse<br>*Community/ Societal*:<br>Athletic or Fraternity membership
Gidycz, Warkentin, Orchowski, and Edwards	2011	414	Sexually aggressive contact	Sexual Experiences Survey, 10 item version (Koss & Oros, 1982)	10.0% (Incidence)	3 months ago	*Individual*:<br>Likelihood of engaging in sex play through arguments or pressure<br>Likelihood of engaging in sex through arguments or pressure<br>Likelihood of engaging in sex through giving alcohol/ drugs
**Sample 3**							
Loh	2003	234<br>*varies slightly depending on risk factor	Sexual aggression	Sexual Experiences Survey, 10 item version (Koss & Oros, 1982)	12.4% (Incidence at 7 month follow up)	3 months ago and 7 months ago	*Individual*:<br>History of perpetration<br>Father/Mother verbal and physical aggression<br>Self-verbal and physical aggression<br>Childhood sexual victimization<br>Alcohol use<br>Rape myth acceptance<br>Hyper gender ideology<br>Adversarial heterosexual beliefs<br>Reactions to Offensive Language and Behavior Scale (comfort and behavior)

							California Psychology Inventory<br>Sexual esteem and preoccupation<br>Sexual- consciousness, motivation, anxiety, assertiveness, depression<br>Eternal/ internal sexual control and Sexual monitoring<br>Fear of sex<br>Sexual satisfaction<br>*Relational*:<br>Token resistance self/ other<br>Socialization (comfort/ behavior)<br>Alcohol expectancy: Self- aggressive, sexual affect, sexual drive, sexual vulnerability, aggressiveness<br>*Community/ societal*:<br>Fraternity membership
Loh, Gidycz, Lobo, and Luthra.	2005	184	Sexual aggression	Sexual Experiences Survey, 10 item version (Koss & Oros, 1982)	12.4% (Incidence at 7 month follow up)	3 months ago and 7 months ago	*Individual*:<br>Perpetration during 3 month follow up<br>Score on Adversarial Heterosexual Beliefs Scale<br>Score on Reactions to Offensive Language and Behavior Scale
**Sample 4**							
Testa and Cleveland	2016	992<br>* FIML techniques to deal with missing data	Sexual aggression perpetration	Sexual Experiences Survey, 16 item version (date not provided)	9.3% (Incidence at Time 5)	Last semester (5 semesters)	*Individual*:<br>Heavy episodic drinking<br>Party attendance<br>Bar attendance<br>Hostility toward women<br>Self-control<br>Antisocial behavior<br>Impersonal sex
**Sample 5**							
Thompson and Morrison	2013	571	Technology- based sexual coercion	Unwanted internet sexual solicitation- participants' sending "sexually suggestive content via texts, emails, or social networking sites during the past year" (p. 5)	21.9% (Incidence)	Last year (4 years and 7 time points)	*Individual*:<br>Anger<br>Impulsivity<br>Sexual compulsivity<br>Hostility towards women<br>Rape supportive beliefs<br>High risk drinking<br>Sexual abuse<br>Pressure for sex<br>Perceived negative sanctions<br>Pornography<br>*Relational*:<br>Interparental conflict<br>Number of sexual partners<br>*Community/Societal*:<br>Peer approval of forced sex<br>Fraternity<br>Varsity sports<br>Student government<br>Religious group

Thompson, Swartout, and Koss	2013	795<br>* FIML techniques to deal with missing data	Sexual aggression	Revised Sexual Experiences Survey (Koss et al., 2007)	16% (Incidence)	Last year (4 years and 7 time points)	*Individual*:<br>Hostile masculinity<br>Alcohol misuse<br>*Relational*:<br>Sex partners<br>*Community/Societal*:<br>Peer norms
Thompson, Kingree, Zinzow, and Swartout	2015	572	Sexual aggression trajectories	Revised Sexual Experiences Survey (Koss et al., 2007)	Statistic not reported but incidence rates used in calculations	Last year (4 years and 7 time points)	*Individual*:<br>Impulsivity<br>Sexual compulsivity<br>Hostility toward women<br>Rape supportive beliefs<br>Heavy drinking<br>Pornography use<br>*Relational*:<br>Number of sexual partners<br>*Community/Societal*:<br>Peer approval of forced sex<br>Peer pressure for sexual activity
Zinzow and Thompson	2015	526	Sexual coercion and assault	Revised Sexual Experiences Survey (Koss et al., 2007)	23% (prevalence during College)	Last year (4 years and 7 time points)	*Individual:*<br>Assault history<br>Risky behaviour<br>Rape supportive believes<br>Antisocial traits<br>Childhood adversity
**Sample 6**							
White and Smith	2004	145<br>*sample size at last time point- no information on how missing data was dealt with	Sexual assault	Sexual Experiences Survey (Koss et al., 1987)	11% (Incidence)	Last year (5 waves separated by a year)	*Individual*:<br>Childhood victimization<br>History of perpetration
White and Smith	2009	136<br>*sample size at last time point- no information on how missing data was dealt with	Sexual aggression perpetration	Sexual Experiences Survey (Koss et al., 1987)	7.7% (Incidence at wave 5)	Last year (5 waves separated by a year)	*Individual*:<br>Prior victimization :<br>Witnessed domestic violence<br>Child victimization<br>Parental punishment
Swartout and White	2010	621<br>*23% of the 621 participants responded to Wave 5	Sexual perpetration experiences	Sexual Experiences Survey (Koss et al., 1987)	Statistic not reported but incidence rates used in calculations	Last year (1 baseline and 4 subsequent waves separated by a year)	*Individual*:<br>Proximal substance use<br>Distal use of marijuana<br>Distal use of other illicit drugs

Walters	2019	851<br>*FIML techniques to deal with missing data	Sexual assault	Set of validated questions from the Longitudinal Study of Violence Against Women	(Incidence)	Last year (5 waves separated by a year)	*Individual*:<br>Previous sexual assault<br>General offending<br>Race<br>*Relational*:<br>Relationship status<br>Blame attributions
**Sample 7**							
Testa, Parks, Hoffman, Crane, Leonard, and Shyhalla	2015	427	Sexual aggression	Questions: (a) "To what extent did you use verbal persuasion to encourage your partner to engage in sexual activity with you?," (b) ". . . use physical pressure or force to encourage your partner to engage in sexual activity with you . . .?," and (c) ". . . encourage her to drink or use drugs as a way of getting her to engage in sexual activity with you?" (p. 509)	Not reported	Last day (56 daily reports)	*Individual*:<br>Hostility toward women<br>Delinquency<br>Socio-sexuality<br>*Relational*:<br>Men's intoxication<br>Type of partner<br>Woman's interest
Testa, Brown, and Wang	2018	298	Sexual aggression	Questions: (a) "To what extent did you use verbal persuasion to encourage your partner to engage in sexual activity with you?," (b) ". . . use physical pressure or force to encourage your partner to engage in sexual activity with you . . .?," and (c) ". . . encourage her to drink or use drugs as a way of getting her to engage in sexual activity with you?" (p. 4)	18.2% (Incidence of use of physical force to obtain sex)	Last day (56 daily reports)	*Individual*:<br>Drinking days<br>*Relational*:<br>Relationship status<br>Drinks 4 hours before sex<br>*Community/societal*:<br>Weekend (vs. weekday)

*There are sixteen articles included in this review across seven distinct samples. In some cases, articles reported on the same sample.
